# Noggin proteins are multifunctional extracellular regulators of cell signaling

**DOI:** 10.1093/genetics/iyac049

**Published:** 2022-03-31

**Authors:** Prashath Karunaraj, Olivia Tidswell, Elizabeth J Duncan, Mackenzie R Lovegrove, Grace Jefferies, Travis K Johnson, Caroline W Beck, Peter K Dearden

**Affiliations:** 1 Laboratory for Development and Regeneration, Department of Zoology, University of Otago, Dunedin 9016, New Zealand; 2 Genomics Aotearoa and Department of Biochemistry, University of Otago, Dunedin 9016, New Zealand; 3 Max Planck Institute for Chemical Ecology, Jena 07745, Germany; 4 School of Biology, Faculty of Biological Sciences, University of Leeds, Leeds LS2 9JT, UK; 5 Biomedical Sciences, University of Tasmania, Hobart, TAS 7005, Australia; 6 School of Biological Sciences, Monash University, Melbourne, VIC 3800, Australia

**Keywords:** Noggins, Noggin-like, bone morphogenic protein, Torso/RTK pathway, dorsal-ventral patterning, terminal patterning, cell signaling evolution

## Abstract

Noggin is an extracellular cysteine knot protein that plays a crucial role in vertebrate dorsoventral patterning. Noggin binds and inhibits the activity of bone morphogenetic proteins via a conserved N-terminal clip domain. Noncanonical orthologs of Noggin that lack a clip domain (“Noggin-like” proteins) are encoded in many arthropod genomes and are thought to have evolved into receptor tyrosine kinase ligands that promote Torso/receptor tyrosine kinase signaling rather than inhibiting bone morphogenic protein signaling. Here, we examined the molecular function of *noggin*/*noggin-like* genes (*ApNL1* and *ApNL2*) from the arthropod pea aphid using the dorso-ventral patterning of *Xenopus* and the terminal patterning system of *Drosophila* to identify whether these proteins function as bone morphogenic protein or receptor tyrosine kinase signaling regulators. Our findings reveal that ApNL1 from the pea aphid can regulate both bone morphogenic protein and receptor tyrosine kinase signaling pathways, and unexpectedly, that the clip domain is not essential for its antagonism of bone morphogenic protein signaling. Our findings indicate that ancestral *noggin/noggin-like* genes were multifunctional regulators of signaling that have specialized to regulate multiple cell signaling pathways during the evolution of animals.

## Introduction 

In animals, cell-to-cell communication depends on a small number of evolutionarily conserved cell signaling receptor superfamilies, 2 of which are the transforming growth factor-β (TGFβ) and receptor tyrosine kinases (RTK) (Babonis and Martindale 2017). These pathways are unique to metazoans and play multiple roles via regulating hundreds to thousands of genes during embryogenesis, pattern formation, and organogenesis (Hill 2001; Furriols and Casanova 2003; Massague and Gomis 2006; Rokas 2008). For cell-signaling pathways to pattern tissues in development, their activation must be tightly regulated, and this occurs via the functions of a relatively small number of regulatory proteins that act in a spatiotemporal manner during development (Carroll 2001). Despite the deep conservation of these signaling pathways, components are absent in some branches of the animal kingdom or have completely different activities in different animals to generate distinct cellular outcomes (Pires-daSilva and Sommer 2003; Babonis and Martindale 2017).

Noggin, a cysteine knot protein, is an extracellular regulator of the TGFβ/bone morphogenic protein (BMP) cell signaling pathway (Zimmerman *et al.* 1996; Avsian-Kretchmer and Hsueh 2004). Noggin was first described in the African Clawed Frog *Xenopus laevis*, where it is secreted from Spemann’s organizer and antagonizes BMP signaling. This activity generates a morphogen gradient along the dorsal-ventral axis, causing prospective ventral mesoderm to become dorsal mesoderm (dorsalization) and prospective epidermis to become neuroectoderm (neutralization; Smith and Harland 1992; Smith *et al.* 1993; Fang *et al.* 2000). Noggin may also interact with activin and wnt in head and limb development (Bernatik *et al.* 2017). In addition to Noggin, many metazoan species possess related “Noggin-like” proteins, which lack a conserved clip domain and have variable sequence length (Molina *et al.* 2011). Noggin-like functions have not been extensively studied but seem to have different functions than those of Noggins.

While *noggin (nog)* genes are present in most animal genomes, they were thought to be absent from insects (Skelly *et al.* 2019). Genome sequencing of hemipteran insects, such as the pea aphid (*Acyrthosiphon pisum*), first revealed genes similar to *nog* and *noggin-like (nog-like)* (Shigenobu *et al.* 2010). Since then, several *nog/nog-like* genes have been identified in other arthropod genomes though not in holometabolous insects (Skelly *et al.* 2019). Phylogenetic analysis indicates that arthropod Noggin/Noggin-like proteins are closely related to 2 well-known signaling proteins in insects, Trunk and Prothoracicotropic hormone (PTTH; Duncan *et al.* 2013). In *Drosophila*, Trunk and PTTH proteins regulate embryonic terminal patterning (Casanova 1990) and the timing of developmental transitions (McBrayer *et al.* 2007), respectively; however, unlike vertebrate Noggins, they act as ligands for RTK signaling. Trunk and PTTH bind to and activate the Torso RTK, triggering signaling via the mitogen-activated protein kinase (MAPK) pathways (Casali and Casanova 2001; Rewitz *et al.* 2009), rather than interacting with morphogenetic proteins. PTTH is also reported to interact with G-protein coupled (GPCR) receptors (Nagata *et al.* 2006; Yamanaka *et al.* 2008), implying it may also signal through G-proteins.

Within the Noggin/Noggin-like/Trunk/PTTH family of proteins (all similar cysteine knot proteins), at least 2 very different signaling activities occur. Canonical Noggins and some Noggin-like proteins function in BMP signaling, while Trunk and PTTH activate RTKs. This implies that this family of proteins has either switched their function entirely from BMP repression to MAPK activation in the insect lineage or that the ancestral Noggin/Noggin-like proteins may be multifunctional and have both MAPK and BMP signaling activity. To distinguish between these possibilities, we focused on understanding the effect on BMP and MAPK signaling of insect *nog/nog-like* genes. Here, we show the outcome of expressing insect *A. pisum nog/nog-like* genes during *Xenopus* dorsoventral (DV) patterning and *Drosophila* terminal patterning. Our findings suggest that pea aphid Noggin-like 1 can repress BMP signaling in *Xenopus* DV patterning and activate MAPK pathway in *Xenopus* animal caps, while pea aphid Noggin-like 2 only activates MAPK pathway in *Xenopus* animal caps and *Drosophila* terminal patterning. These findings support a model whereby ancient extracellular regulators could have been a multifunctional protein, which was later co-opted into other developmental processes, losing its primary role.

## Materials and methods

### Molecular cloning

The clip domain of *ApNL1* (encoding the amino acids PVPSNDPGVIDLIEMP) was deleted from *ApNL1* to synthesize *ApNL1*Δ*Clip* and inserted into *ApNL2* and *Drosophila melanogaster trunk* (35–47 and 47–63 aa residues, respectively) to synthesize *ApNL2+Clip* and *DmTrk+Clip*. The candidate genes (Supplementary Table 1) were synthesized by GenScript in pBluescript (KS) vector. Genes were subcloned into pCS2 vector for generation of capped polyadenylated mRNA for injection into *Xenopus* oocytes. Genes were subcloned into pUASP attB vector for the generation of transgenic fly lines.

### *Xenopus* injections

*Xenopus* embryos were generated and cultured (University of Otago Animal Use Protocol 19/01) as described previously (Beck and Slack 1999). *Xenopus* embryos were staged according to Nieuwkoop and Faber (1994). mRNA for injection was generated from linearized plasmid using the SP6 mMESSAGE mMACHINE kit (Ambion). Dorsal-anterior index (DAI) scores (Kao and Elinson 1988) were recorded at stage 32. A score of 5 indicated a wild-type phenotype, with lower scores down to 0 indicating the degree of centralization and higher scores up to 10 indicating the degree of dorsalization.

### Animal cap assay

**Fig. 1.**
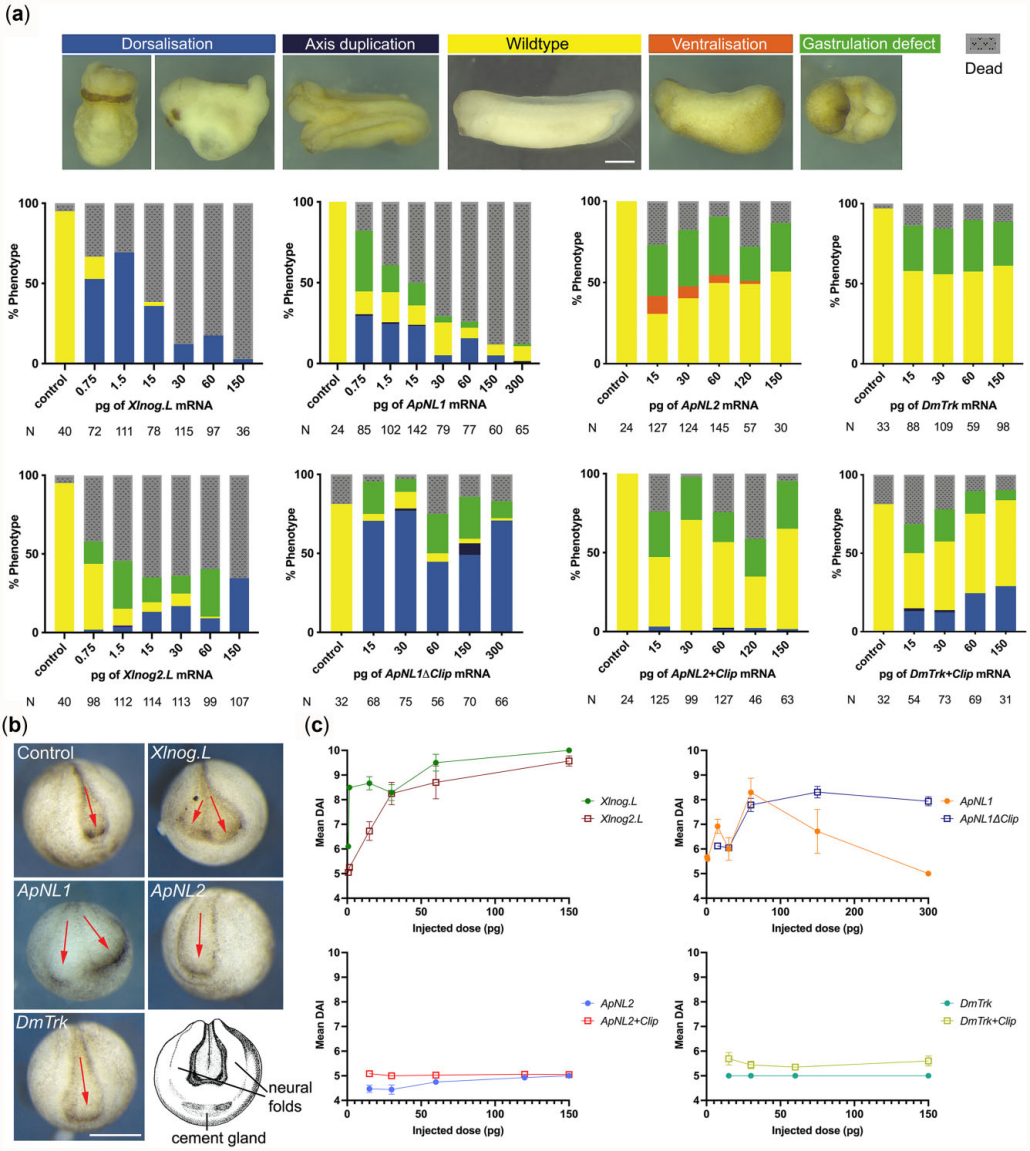
ApNL1 induces dorsalization in *Xenopus* embryos. a) Phenotypes observed at stage 32 *Xenopus* embryos for microinjections of *Xlnog.L*, *Xlnog2.L*, *ApNL1*, *ApNL1*Δ*Clip*, *ApNL2*, *ApNL2+Clip*, *DmTrk*, and *DmTrk+Clip*. Controls were injected with dH_2_O. N- total *Xenopus* embryos injected. b) Anterior view of stage 16 *Xenopus* embryos show presence/absence of axis duplication. c) Mean DAI charts for the microinjections of *Xlnog.L*, *Xlnog2.L*, *ApNL1*, *ApNL1*Δ*Clip*, *ApNL2*, *ApNL2+Clip*, *DmTrk*, and *DmTrk+Clip.* Error bars denote SEM. DAI <5—ventralization; DAI 5- wild type: DAI >5—dorsalization. See also Supplementary Fig. 2. Scale bar 1 mm.

The dose for mRNA injection was selected based on the *Xenopus* functional screening data (Fig. 1, see also Supplementary Fig. 2): *XlNog1*—5.6 pg, *ApNL1*—1.5 pg, *ApNL2*—30 pg, *DmTrk*—60 pg. The final concentration of human bFGF (Sigma-Aldrich) is 100 ng/ml. Animal caps were dissected as previously described (Ariizumi *et al.* 2017) and placed at 18°C. *N* = 15+ caps for control, FGF, and each mRNA injection.

### Histological analysis

Animal caps were resin-embedded using Technovit 7100 protocols (Kulzer Technik). After 4 days of polymerization, the embedding molds were removed by placing the embedding tray at 60°C for 5 min. Next, animal caps were sectioned at 2 µm using a microtome (Reichert-Jung). The sections were then placed on a water bath at 25°C and transferred to microscope slides, later dried at 60°C for an hour. Microscopic slides were flooded with Polychrome I (Sigma-Aldrich) for 60 s and washed with running water. Then the slides were flooded with Polychrome II (Sigma-Aldrich) for 30 s and later washed with running water, followed by a few dips in 95% ethanol and washing with running water. The slides were then dried in a fume hood. Entellan rapid mounting medium (Merck Millipore) was applied to dried slides, and coverslips were placed on them. The slides were left overnight to dry and imaged using an Olympus BX51 microscope.

### RT-PCR

For each sample, 8 animal caps were pooled into a 2 ml micro-centrifuge tube. RNeasy Mini Kit (Qiagen) was used to isolate genomic RNA (gRNA) from the animal caps. Isolated RNAs were then stored at −80°C. The amount of gRNA used in reverse transcription was consistent for each sample (150 ng). The mixture for first cDNA synthesis consisted of 150 ng of template RNA, 2 μl of Random Primers (Invitrogen), 2 μl of 10 × M-MuLV buffer (NEB), 1 μl of M-MuLV Reverse transcriptase (NEB), 1 μl of 10 mM dNTPs (Invitrogen), 0.2 μl of RNase inhibitor (Invitrogen) and the volume was made up to 20 μl with dH_2_O. The mixture was then placed in a thermocycler (Eppendorf), and the following incubation periods were set up to run: 25°C for 5 min, 42°C for an hour, 65°C for 20 min. The concentrations of cDNA samples were measured using a Nanodrop2000 spectrophotometer and then stored at −20°C. Mesoderm and neural markers (Supplementary Table 2) were selected from the previous studies (Reilly and Melton 1996; Sakata and Maeno 2014).

### *Drosophila* stocks

The following *Drosophila* stocks were used: Oregon-R (BL5), *GAL4::VP16.nos.UTR* (BL7253) and *trk*^Δ^*—*a null mutant of *trunk* (Henstridge *et al.* 2014). Germline transformants of *Drosophila* were obtained by microinjection of pUASP plasmid containing the gene of interest into PhiC31 source *Drosophila—*attP docking site 86F6 (24749) (BestGene Inc). Ectopic expression was driven in the adult ovary by crossing pUASP lines (Supplementary Table 3) with the *GAL4::VP16.nos.UTR* (BL7253) driver line and *trk*^Δ^*^;^ GAL4::VP16.nos.UTR* driver lines. The flies were maintained, and crosses were set up at 25°C.

### In situ hybridization chain reaction and cuticle preparation

*Drosophila* adults were allowed to lay eggs on apple juice plates containing yeast paste for 4 h before being removed for in situ hybridization chain reaction (HCR) for *Dm-tailless (tll)* as described previously (Choi *et al.* 2016). Embryos were imaged using an Olympus FV1000 confocal microscope. Cuticle preparations were prepared by mounting dechorionated *Drosophila* embryos in Hoyer’s medium using established methods (Henstridge *et al.* 2014) and visualized using a darkfield modified Olympus BX61 microscope.

### Immunostaining and TUNEL assay for *Drosophila* ovarioles

Flies (2 days old) were left for 24 h in fly food containing Baker’s yeast before dissection. *Drosophila* ovaries were dissected in 1 × PBS and placed into 400 μl of ice-cold 1 × PBS. The ovaries were fixed with 100 μl of 37% formaldehyde and 500 μl heptane by placing them on a nutator for 20 min. Ovaries were then washed with 1 × PBS for 3 times 5 min each on a rocker. Immunostaining for cleaved *Drosophila* death caspase 1 (Asp216) (Cell Signaling Technology) was performed using the established protocol (Luo *et al.* 2013). TUNEL assays with BrdU-Red (Abcam-ab66110) were performed using the manufacturer’s protocol. The ovarioles were separated using tungsten needles and visualized using an Olympus FV3000 confocal microscope.

## Results

### *Acyrthosiphon pisum* Noggin-like1 acts as a BMP antagonist in *Xenopus* embryos

In *Xenopus*, BMP signaling is blocked locally by Noggin, leading to dorsal development (anterior dorsal structures, i.e. forebrain, cement gland, eye, and neural ectoderm). If *noggin* is overexpressed in *Xenopus* embryos, it induces dorsal structures in embryos or induces a secondary axis when ventrally expressed (Fang *et al.* 2000; Molina *et al.* 2011). We used this assay to determine if *nog/nog-like* genes from *A. pisum* [*nog-like 1* (*ApNL1*) and *nog-like 2* (*ApNL2*), Supplementary Fig. 1] are able to antagonize BMP signaling activity. To do this, we injected mRNA from these genes into *Xenopus* embryos at the 2-cell stage and quantified dorsalization using the morphological dorsal-anterior index (DAI; Kao and Elinson 1988) at stage 32. Each injected mRNA causes differing degrees and amounts of phenotypes indicating different functions for each protein and different degrees of activity. We began injections with the same amounts of mRNA, but higher doses of some mRNAs were lethal. Micro-injection of *ApNL1* dorsalized *Xenopus* embryos consistent with the dorsalizing effects of micro-injection of *X.* *laevis noggin. L* and *noggin2.L* (*Xlnog2.L*) but also produced gastrulation defects, not typical of *noggin* overexpression (Fig. 1a). Injection of either *ApNL1* or *Xlnog.L* mRNA also caused lethality. In contrast, *ApNL2* and *D.* *melanogaster trunk* (*DmTrk*) did not dorsalize embryos and instead produced high frequencies of gastrulation defects (>25%), where embryos failed to close their blastopores (Fig. 1a).

Axis duplication, a dorsalization phenotype, can be detected in the early developmental stages of *Xenopus* (stage 16/mid-neurulation) in the event of dorsalization (Glinka *et al.* 1997). Axis duplications were frequently observed in *Xlnog.L* and *ApNL1* injected embryos but never in *ApNL2* and *DmTrk* injected embryos (Fig. 1b). Ectopic expression of *Xlnog.L*, *Xlnog2.L*, and *ApNL1* resulted in mean DAI scores >5 (dorsalized), increasing with dose (Fig. 1c, see also Supplementary Fig. 2) [those embryos that survived a high dose (300 pg) of *ApNL1* are wild type, suggesting all properly injected embryos died]. Mean DAI for *ApNL2* or *DmTrk* injected embryos never exceeded 5 (Fig. 1c, see also Supplementary Fig. 2). This evidence suggests *ApNL1* encodes a BMP antagonist and is an arthropod *noggin*, while *ApNL2* is not.

### The clip domain of *ApNL1* is not essential for BMP inhibition

Crystallographic analysis of the interaction between human Noggin (hNoggin) and BMP-7 showed that the clip domain (Gln 28 to Glu 48) of hNoggin mediates its binding to BMP-7, and *in vivo* studies of substitution mutations at Pro35, Leu46, and Glu48 of hNoggin abolish this interaction (Groppe *et al.* 2002). To examine whether this clip domain is needed for BMP inhibition by ApNL1, we deleted 15 amino acid residues (PVPSNDPGVIDLIEMP) from ApNL1 (ApNL1ΔClip) and inserted them into ApNL2 (ApNL2+Clip) and DmTrk (DmTrk+Clip) (Supplementary Fig. 1). Intriguingly, *ApNL1*Δ*Clip* mRNA induced *Xenopus* embryo dorsalization but was tolerated at higher doses (>30 pg) compared to *ApNL1* injections (Fig. 1a), implying that ApNL1ΔClip protein has a weaker BMP inhibiting activity. These data imply that the arthropod Noggin ApNL1 has an inhibitory action on BMP via a molecular mechanism that does not solely require the clip domain. Introducing the clip domain into the *D. melanogaster trunk* and *ApNL2* yielded small but notable numbers of dorsalized embryos (Fig. 1a). As the introduction of a clip domain into *trunk* confers BMP antagonism, it is clear that the clip domain also contributes to BMP inhibition.

### Both ApNL1 and ApNL2 activate RTK/MAPK pathway in *Xenopus* animal caps

**Fig. 2.**
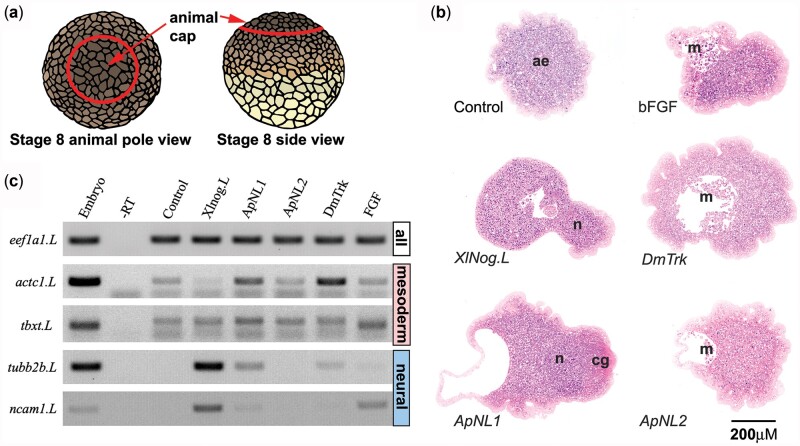
*ApNL1* and *ApNL2* induce mesoderm in *Xenopus* animal caps. a) Schematic of animal cap explants. b) Representative sections of resin-embedded sections of Polychrome stained animal cap explants. Controls were injected with dH_2_O. Basic FGF (bFGF) protein was used as a positive control for MAPK activity. ae, atypical epidermis; n, neural; *cg*, cement gland (neural), *m*, mesenchyme (ventral mesoderm). Scale bar 200 µm. c) RT-PCR of mesodermal (*actc1.L* and *tbxt*. *L*) and neural (*tubb2b.L* and *ncam1.L*) markers. The negative control is genomic RNA from *Xenopus* embryo without Reverse Transcriptase (RT) enzyme. Ubiquitously expressed elongation factor 1-α (*eef1a1.L*) is the loading control.

Duncan *et al.* (2013) suggested that the arthropod-specific genes *trunk* and *PTTH* evolved from an ancestral arthropod *nog*/*nog-like* gene. If these ancestral arthropod Noggin/Noggin-like proteins were also RTK/MAPK activators, like PTTH and Trunk, this activity might also be conserved in extant Noggin/Noggin-like proteins. To investigate whether arthropod *A. pisum* Noggin-like proteins can activate the MAPK signaling pathway in *Xenopus*, animal cap assays (Yamada and Takata 1961; Ariizumi *et al.* 2009) were performed (Fig. 2, a and b). When cultured in a simple salt solution, the animal cap, an area around the animal pole of mid-blastula stage *Xenopus* embryos, forms an irregularly shaped epidermis, called the atypical epidermis (Yamada and Takata 1961; Ariizumi *et al.* 2009). However, activinA (Ariizumi *et al.* 1999) and *Xenopus* Noggin (Lamb *et al.* 1993; Reversade and De Robertis 2005) can induce neural tissues in animal caps by suppressing BMP signaling, while basic fibroblast growth factor (bFGF) mediates mesodermal differentiation in animal caps via the MAPK pathway (LaBonne and Whitman 1994; Umbhauer *et al.* 1995).

Injections of canonical Noggin *Xlnog.L* mRNA induced neural fate in animal caps (14/14) as confirmed by RT-PCR for neural markers (*tubb2b.L* and *ncam1.L*, Fig. 2, Table 1), consistent with BMP antagonism (Lamb *et al.* 1993). bFGF (100 ng/ml), an RTK ligand, induced mesenchyme (4/16), part of the ventral mesoderm (Umbhauer *et al.* 1995), in animal caps (Table 1) and the expression of mesodermal markers [*actc1.L*, expressed in muscle (ventral mesoderm) (Gotoh *et al.* 1995) and *tbxt*. *L* (*brachyury*) in pan mesoderm] (Cordenonsi *et al.* 2007) as confirmed by RT-PCR. Injection of *DmTrk* also induced mesenchyme (17/20) and expression of the mesoderm marker (*actc1.L*), indicating that it can activate MAPK signaling activity in *Xenopus* animal caps, plausibly via *Xenopus* RTKs (Table 1, Fig. 2c). Microinjection of *ApNL1* produced neural tissue (10/21) and cement gland (7/21), consistent with BMP repression, but also produced mesenchyme (9/21, Table 1). RT-PCR confirmed this for neural markers (*tubb2b.L* and *ncam1.L*) and mesoderm markers (*actc1.L* and *tbxt*. *L*, Fig. 2). Microinjection of *ApNL2* mRNA generated mesenchyme (18/18, Table 1) and expression of *tbxt*. *L* (pan mesoderm marker). Our findings imply that when overexpressed, ApNL1 may both **repress** BMP signaling and **activate** the MAPK pathway, while ApNL2 and Trunk only activate the MAPK pathway in *Xenopu*s animal caps.

**Table 1.** Observed tissue types for each sample.

		Types of cells/tissues observed (number of caps)				
		Epidermis	Neural tissue	Cement gland	Mesenchyme	Atypical epidermis
*mRNA* injected (number of caps)	Control (9)	—	—	—	—	9
	*Xlnog.L* (14)	7	14	—	—	—
	*ApNL1* (21)	21	10	7	9	—
	*ApNL2* (18)	18	—	—	18	—
	*DmTrk* (20)	20	6	4	17	—
	FGF (16)	16	—	—	4	—

### ApNL2 activates Torso/MAPK pathway in *Drosophila* terminal patterning

Next, we wanted to analyze whether arthropod Noggin-like proteins can act as RTK ligands in *Drosophila* terminal patterning—a robust model system to study the Torso/MAPK pathway (Lu *et al.* 1993), or can inhibit Decapentaplegic (Dpp), a BMP-2/4 ortholog (Kawabata *et al.* 1998), in *Drosophila* DV patterning. We generated *nog/nog-like* transgenes and performed maternal over-expression using *nos*-Gal4 driver lines (Wang *et al.* 1994) and rescue assays using a *trunk* null (*trk*^Δ^) mutant background (Henstridge *et al.* 2014) during *Drosophila* oogenesis and embryogenesis.

**Fig. 3.**
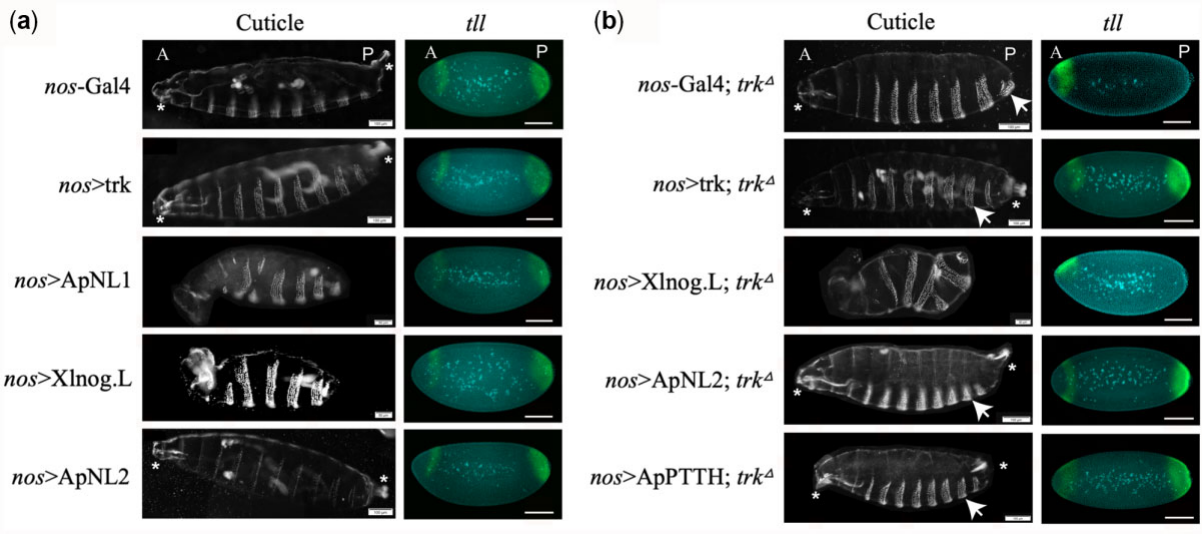
ApNL2 rescues *trk*^Δ^ mutant phenotypes while *Xlnog.L* ventralizes *Drosophila* embryos. a) Maternal expression of genes expressed via the *nanos* (*nos*) promoter during *Drosophila* embryo patterning. Wild-type control (*nos*-Gal4) first instar larvae cuticle shows normal terminal structures and normal *tailless* (*tll*) expression in stage 4 embryos. b) Maternal expression of genes in *trunk* null mutant flies during *Drosophila* terminal patterning. The *trunk* null mutant (*nos*-Gal4; *trk*^Δ^) cuticle lacks abdominal segment after A7 (arrowed) and posterior *tll* expression in stage 4 embryos. DAPI—staining in centre of embryos, *tll*—staining at the terminal ends of the embryos. A, anterior; P, posterior. Anterior mouth-hooks and posterior filzkörper are marked by asterisks. Scale bar 100 µm or otherwise stated.

Maternal over-expression of *DmTrk* during *Drosophila* embryo patterning did not produce expanded terminal structures in cuticles (240/240), nor cause broadening of the terminal *tailless* (*tll*) expression domain (Fig. 3a) as expected since the Torso receptor activation in *Drosophila* is a rate-limiting step and regulated by Torso-like at the posterior end (Stevens *et al.* 1990). Maternal over-expression of *ApNL1* and *Xlnog.L* during *Drosophila* embryo patterning ventralized *Drosophila* embryos at the expense of anterior and posterior terminal structures (14/16 and 204/209, respectively, Fig. 3a, note loss of the terminal mouth-hooks and filzkörper), an observation consistent with previous studies of *Xlnog.L* (Holley *et al.* 1996), implying that they both inhibit Dpp during *Drosophila* DV patterning. Intriguingly, *nos*>*ApNL1* female flies laid far fewer eggs than females expressing other constructs. In addition, *ApNL1* partially suppressed anterior *tll* expression in stage 4 embryos, implying that *ApNL1* may interfere with the activation of Torso/MAPK pathway, perhaps by dimerizing with endogenous trunk and antagonizing its activity. In contrast, over-expression of *ApNL2* during *Drosophila* embryo patterning did not alter either terminal structures of first instar larval cuticles (282/282) or *tll* expression (Fig. 3a). When we attempted to rescue the terminal phenotypes of *trk*^Δ^ mutants by over-expressing *noggin* and *noggin-like* genes using *nos*-Gal4 drivers, we again observed strong ventralization by *Xlnog.L* (71/71) without any rescue of *tll* expression (Fig. 3b). We did not observe any embryos for the *trk*^Δ^ rescue assay of *ApNL1* as the female flies did not lay eggs. In contrast, both *ApNL2* and *ApPTTH* rescued *trk*^Δ^ mutant terminal phenotypes (72/72 and 53/53, respectively) and *tll* expression (Fig. 3b), indicating that these proteins act as Torso ligands.

### Maternal over-expression of *ApNL1* triggers apoptosis during *Drosophila* oogenesis

**Fig. 4.**
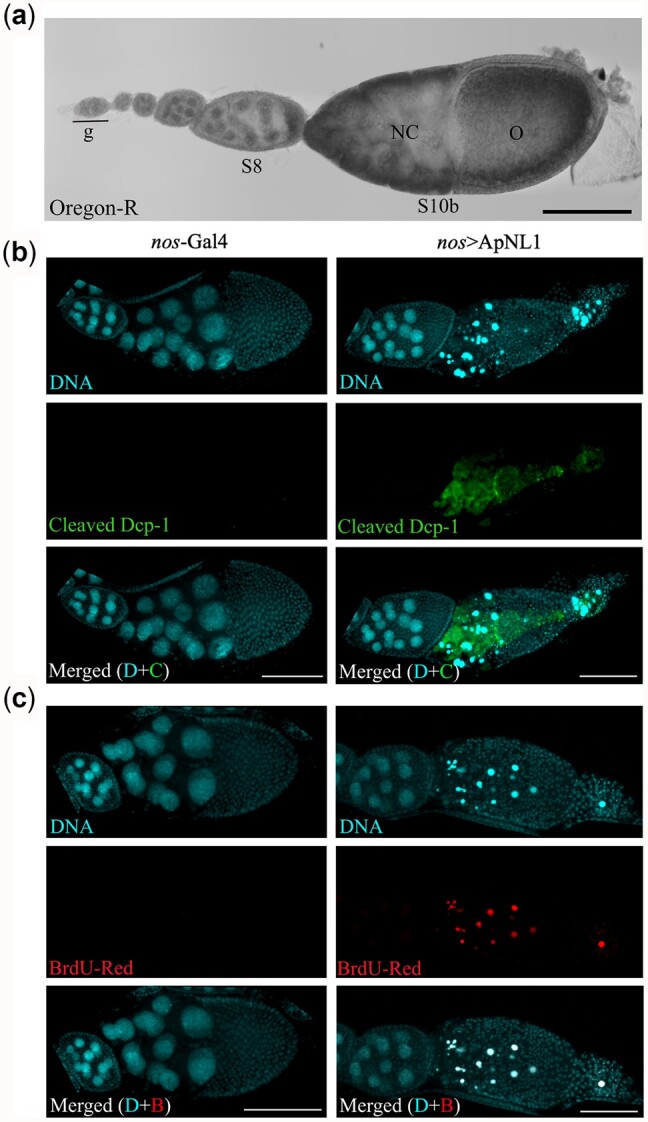
Maternal expression of *ApNL1* triggers apoptosis during *Drosophila* oogenesis. a) A single ovariole of wild-type Oregon-R fly strain. g, germarium; NC, nurse cells; O, oocyte. b) Immunostaining for cleaved *Drosophila* death caspase 1 (Dcp-1) during late *Drosophila* oogenesis of *nos*-*Gal4* and *nos*>*ApNL1* female flies. DAPI—cyan, cleaved Dcp-1—green. c) TUNEL assay with BrdU-Red for late stages of *Drosophila* oogenesis of *nos*-Gal4 and *nos*>ApNL1 female flies. Scale bar 100 µm.

When we expressed *ApNL1* in *Drosophila* maternal tissues, *nos>*ApNL1 female flies laid fewer eggs (*n* = 16) than females from other crosses and none in our *trk*^Δ^ rescue assay. Examination of *nos>*ApNL1 female ovarioles revealed the arrest of stage 9 egg chambers during oogenesis (Fig. 4), a phenotype reminiscent of reduced Dpp signaling (Twombly *et al.* 1996). Furthermore, immunostaining revealed the presence of the apoptosis marker Dcp-1 (cleaved *Drosophila* death caspase 1) (85/85, Fig. 4b) and positive TUNEL staining (54/54, Fig. 4c), in stage 9 egg chambers of *nos*>ApNL1 female flies, indicating that maternal *ApNL1* expression induces cell death in late oogenesis. Given that ApNL1 ventralizes *Drosophila* embryos (Fig. 3a), likely through interfering with Dpp activity, it is plausible that *ApNL1* expression also disrupts Dpp signaling during *Drosophila* oogenesis.

## Discussion

The relationship between Noggin proteins, which act to repress BMP signaling, and Trunk/PTTH proteins, which activate MAPK, implies that the function of these proteins has switched in their evolution. The evolution of Trunk/PTTH, presumably from a Noggin/Noggin-like ancestor, involved loss of the clip domain, loss of BMP inhibitory activity, and gain of interaction with Torso. In nonholometabolous insect genomes, a number of Noggin and Noggin-like proteins seem to be present, sometimes with Trunk/PTTH proteins as well (Skelly *et al.* 2019). Here, we show that 2 of these genes in *A. pisum* have different molecular functions, with ApNL1 acting to inhibit BMP/Dpp signaling and activate MAPK, and ApNL2 acting to activate MAPK/RTK signaling, similar to Trunk and PTTH.

The clip domain, which has been shown to be required for BMP inhibition by hNoggin (Groppe *et al.* 2002), is not required for inhibition by ApNL1, indicating that some other part of the protein carries out this function. It is possible that BMP inhibition by the clip domain in hNoggin is an evolutionary specialization. The noninhibitory function of the clip domain in ApNL1 may also relate to the very strong BMP inhibition by the protein. In *Drosophila* over-expression of ApNL1 causes phenotypes in the embryo and ovary, whereas over-expression of Xnog.L does not. It is possible this stronger inhibition of BMP by ApNL1 reflects the fact that it is carried out by a part of the protein other than the clip domain.

That ApNL1, while strongly inhibiting BMP, also has weak MAPK activity in *Xenopus* implies that this protein may be multifunctional and that the evolutionary history of Noggins and Trunk/PTTH is not a switch from one form of signaling to another, but the specialization of an ancestrally multifunctional protein into either regulating BMP or MAPK signaling. Noggin/Noggin-like proteins have also been shown to have activities on wnt signaling pathways (Eroshkin *et al.* 2016), and PTTH on GPCRs (Nagata *et al.* 2006; Yamanaka *et al.* 2008), implying that Noggin/Noggin-like ancestrally may have been multifunctional extracellular regulators of multiple cell signaling pathways.

## Data availability

All data for this study are presented in the manuscript or in the Supplementary data.

Supplemental material is available at *GENETICS* online.

## Supplementary Material

iyac049_Supplementary_Figs-Table

iyac049_Supplemental_Mateiral_Legend
