# Supplementary material for: Noggin proteins are multifunctional extracellular regulators of cell signaling
Source: Genetics. 2022 Mar 31;221(1):iyac049. doi: 10.1093/genetics/iyac049 (PMC9071555; doi:10.1093/genetics/iyac049)
Supplement: iyac049_Supplementary_Figs-Table [file iyac049_supplementary_figs-table.pdf]

```
HsNog          1 MERCPS---------LGVTLYALVVVLGLRATPA-----GG----QHYLH--------IR
XlNog.L        1 MDHSQC---------L-VTLYALMVFLGLRIDQG-----GC----QHYLH--------IR
XlNog2.L       1 MKRINL---------P--EAFLLCLWLFLVHHQG-----SC----QPYLR--------LR
ApNL1          1 MTVKWL---------F-----VL-VALTCCSNRH---TVMCTSVRLDHLP--------LR
ApNL1-Clip     1 MTVKWL---------F-----VL-VALTCCSNRH---TVMCTSVRLDHLP--------LR
ApNL2          1 MRRSFD---------V--GVALL-IVLTAVGE-Q-----LL----QHSMA----INSPAK
ApNL2+Clip     1 MRRSFD---------V--GVALL-IVLTAVGE-Q-----LL----QHSMA----INSPAK
DmTrk          1 MFLRILCPTMKSQSELAIVLTWL-AVLGTAQDDAD----YCAELSTQSLAK--------I
DmTrk+Clip     1 MFLRILCPTMKSQSELAIVLTWL-AVLGTAQDDAD----YCAELSTQSLAK--------I
ApPTTH         1 MRPQ---------Y-IHFIRTMH-VVLGSISIWTLYALVSCVSVSCLSMAKKVRWELVYD
DmPTTH         1 MDIKVW---------------RL-LGQSCRRSTA---LVRHRSSMMPWLP--------IS

HsNog         35 ---------------PAPSD----------NLPLVDL-----IEHPDPIFDPKE-KDLNE
XlNog.L       34 ---------------PAPSE----------NLPLVDL-----IEHPDPIYDPKE-KDLNE
XlNog2.L      33 ---------------PSPSE----------NLPVKDI-----IEHPDPEQDPKE-QDLDE
ApNL1         35 ---------------PVPSN----------DPGVIDL-----IEMPNPALDPRP-SDINA
ApNL1-Clip    33 ----------------------------------------------NPALDPRP-SDINA
ApNL2         35 -----------------KT-----------SGESFSV-----WFRAEPG-LPRK-KDMKV
ApNL2+Clip    35 ---------------PVPSN----------DPGVIDL-----IEMPEPG-LPRK-KDMKV
DmTrk         48 ---------------LGQAF----------NPRYMS------IDPPG---EPEE-KSYHL
DmTrk+Clip    48 ---------------PVPSN----------DPGVIDL-----IEMPG---EPEE-KSYHL
ApPTTH        50 ---------------PLPNDESFDRFAAADHHATVNL-----IQQRD---EQQQQRDEQ-
DmPTTH        34 IMAMALLSLTNKGIASQYAS----------DEGIDEMVGLRSLEHRAEEQQP----DRST

HsNog         64 T-LLRSLL---GGHYDPGFMATSP---PEDRPGGGGGAAGGAED-LAELDQLLRQRPSGA
XlNog.L       63 T-LLRTLM---VGHFDPNFMATIL---PEERLGV--------ED-LGELDLLLRQKPSGA
XlNog2.L      62 R-TLRKKL---GSNFDPNFMSVVL---PNSVNTS-------------TQDSLTKMKTLGS
ApNL1         64 T-ALLAKL---AAKFDPNCMSMTA---PENLVPA--------TEIPFRRNRRGRLVPTGK
ApNL1-Clip    48 T-ALLAKL---AAKFDPNCMSMTA---PENLVPA--------TEIPFRRNRRGRLVPTGK
ApNL2         60 G-KLNRLI---GADYNEMWMSKTAIVITEDHTN-----------------------AS
ApNL2+Clip    63 G-KLNRLI---GADYNEMWMSKTAIVITEDHTN-----------------------AS
DmTrk         73 G-YKRSS-------YELP---FYA---DSDAISV--------SHFPAWETNHFALVEKKK
DmTrk+Clip    74 G-YKRSS-------YELP---FYA---DSDAISV--------SHFPAWETNHFALVEKKK
ApPTTH        86 ------------------------------------------------------------
DmPTTH        80 SKMLSALFGFSPSTPHEPTEMAMVM---PHQLPPM--------YYNDFYE----DLVTTKR

HsNog        116 MPSEIKGLEFSEG---LAQGKKQRLSKKLRRKLQMWL-----------------------
XlNog.L      107 MPAEIKGLEFYEG---L-QSKKHRLSKKLRRKLQMWL-----------------------
XlNog2.L     102 IPLELKKLDLSET---P-YGGRIRMGKKARRKFLQWL-----------------------
ApNL1        109 MPTEIRELDTKYFFLSDGRRLRTRITGQLRRKIQQYL-----------------------
ApNL1-Clip    93 MPTEIRELDTKYFFLSDGRRLRTRITGQLRRKIQQYL-----------------------
ApNL2         91 GAADVKSLETAGP---DNLQLPEQLPAQYQEMMRTWL-----------------------
ApNL2+Clip    94 GAADVKSLETAGP---DNLQLPEQLPAQYQEMMRTWL-----------------------
DmTrk        111 EAPRSKSLR----------------------------TRSAFMDRVGHPRIDGFK-----
DmTrk+Clip   112 EAPRSKSLR----------------------------TRSAFMDRVGHPRIDGFK-----
ApPTTH        86 ---QQWRHR---------------------EEQQQWRYRNEQQQRQHHHQQQQKRDEWQ
DmPTTH       125 --------------------------------NDVH------------------------

HsNog        150 ---WSQT---------FCPVLYAWNDLGSRFWPRYVKVGSCFSK---RSCSVPEGMVCKP
XlNog.L      140 ---WSQT---------FCPVLYTWNDLGTRFWPRYVKVGSCYSK---RSCSVPEGMVCKA
XlNog2.L     135 ---WAYT---------YCPVMYTWKDLGVRFWPRFIKEGHCFSE---KSCSFPEGMYCKP
ApNL1        146 ---WGRT---------ACPMHRQWKDLGQRFWPRWLLEGHCPKGE--TSCSVPAGMYCRA
ApNL1-Clip   130 ---WGRT---------ACPMHRQWKDLGQRFWPRWLLEGHCPKGE--TSCSVPAGMYCRA
ApNL2        125 ---VHRS---------KCPVEYAWADLGKSFWPRWIKKGRCGRSET-MSCSWPPGMSCMP
ApNL2+Clip   128 ---VHRS---------KCPVEYAWADLGKSFWPRWIKKGRCGRSET-MSCSWPPGMSCMP
DmTrk        138 ---QRPW---------ECSSKINWIDLGLNYFPRYIRSIECIAR----KC-WYDHFNCKP
DmTrk+Clip   139 ---QRPW---------ECSSKINWIDLGLNYFPRYIRSIECIAR----KC-WYDHFNCKP
ApPTTH       122 QLRYADTLSVAASEACNCTEVPLVYNLTENHYPKELMSVTC------------SGNLCKT
DmPTTH       129 ---SAGC---------DCKVTNELVDLGGLHFPRFLMNAVCESGAGRDLAKCSHGSNCRP
```

```
HsNog        195 SKSVHLTVLRWRCQRRGG-----------------------------------------Q
XlNog.L      185 AKSMHLTILRWRCQRRVQ-----------------------------------------Q
XlNog2.L     180 IKSVTKTFLRWYCQGWTR----------------------------------------QK
ApNL1        192 TSSQYKTLLRWHCRSPNGPSSVLQQY---NSLPGAKSTGSV--------------VNQIK
ApNL1-Clip   176 TSSQYKTLLRWHCRSPNGPSSVLQQY---NSLPGAKSTGSV--------------VNQIK
ApNL2        172 SSIRVLNLLRWHCWLKNRKNKRKNRERKTMELAFNKKRGHAARGRRAAGQRKSKKKDDKF
ApNL2+Clip   175 SSIRVLNLLRWHCWLKNRKNKRKNRERKTMELAFNKKRGHAARGRRAAGQRKSKKKDDKF
DmTrk        181 KSFTIKVLRRKTGSCIRIN--------------------------DKLILITAEKFENDY
DmTrk+Clip   182 KSFTIKVLRRKTGSCIRIN--------------------------DKLILITAEKFENDY
ApPTTH       170 AWYSVPVLLKSNTQQVENQ--------------------------DDLP----DE-LQQN
DmPTTH       177 LEYKVKVLAQT-S-QSDHPYS----------------------------------W-MNK

HsNog        214 RCGWIPIQYPIISECKCSC------
XlNog.L      204 KCAWITIQYPVISECKCSC------
XlNog2.L     200 YCTWIPVQYPIISECKCSC------
ApNL1        235 VCQWIKVEYPVVTECGCGCATDVSE
ApNL1-Clip   219 VCQWIKVEYPVVTECGCGCATDVSE
ApNL2        232 RCLWIKVPYPVTEDCTCSCKK-PDE
ApNL2+Clip   235 RCLWIKVPYPVTEDCTCSCKK-PDE
DmTrk        215 TQLWIWEEIAVNFCCECVML----Y
DmTrk+Clip   216 TQLWIWEEIAVNFCCECVML----Y
ApPTTH       199 VNHWKFDSVNITVACYCSI-----K
DmPTTH       200 DQPWQFKTVTVT--AGCFCTK----
```

Figure S1: Multiple sequence alignment of Noggin, Noggin-like, Trunk and PTTH proteins with human Noggin. Essential residues in Clip domain of hNoggin - cyan. 15 aa residues of ApNL1- magenta. Deletion and insertion in constructs- red. Hs- *Homo sapiens*, Xl- *Xenopus laevis*, Ap- *Acyrthosiphon pisum*, Dm- *Drosophila melanogaster*. Nog- Noggin, NL- Noggin-like, Trk- Trunk, PTTH- Prothoracicotropic hormone.

A

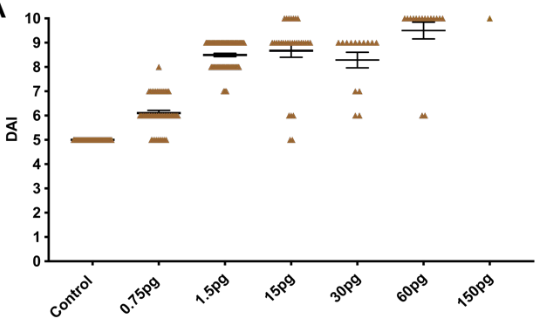

N	38	48	77	30	14	16	1
Mean	5	6.104	8.494	8.667	8.286	9.5	10
SD	0	0.7506	0.5988	1.47	1.204	1.366	0
SEM	0	0.1083	0.06823	0.2684	0.3219	0.3416	0
ANOVA	--	****	****	****	****	****	****

B

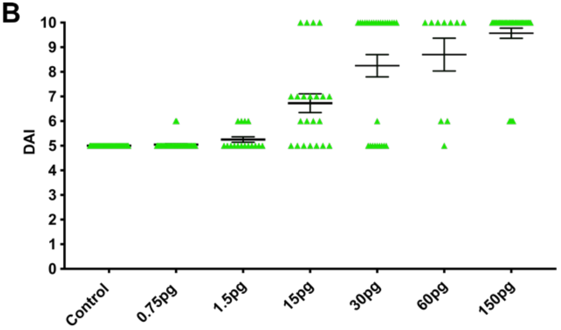

N	38	43	16	22	28	10	37
Mean	5	5.047	5.25	6.727	8.25	8.7	9.568
SD	0	0.2131	0.4472	1.778	2.398	2.111	1.259
SEM	0	0.03249	0.1118	0.379	0.4532	0.6675	0.207
ANOVA	--	ns	ns	****	****	****	****

C

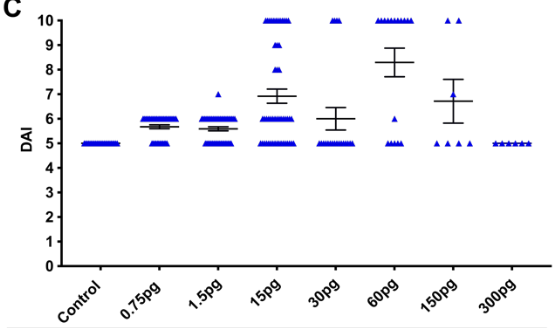

N	24	37	44	50	20	17	7	6
Mean	5	5.676	5.591	6.92	6	8.294	6.714	5
SD	0	0.4746	0.5421	2.049	2.052	2.392	2.36	0
SEM	0	0.07802	0.08172	0.2897	0.4588	0.5801	0.8921	0
ANOVA	--	ns	ns	****	ns	****	*	ns

D

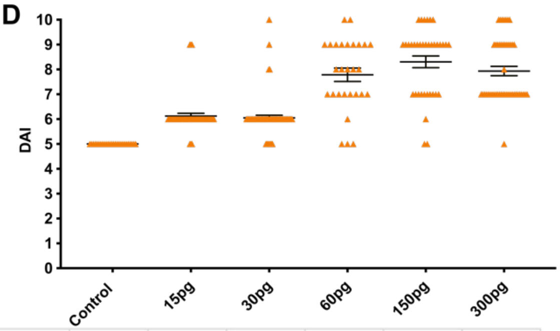

N	26	49	61	28	36	47
Mean	5	6.122	6.049	7.786	8.306	7.936
SD	0	0.7808	0.8252	1.424	1.411	1.275
SEM	0	0.1115	0.1056	0.269	0.2351	0.186
ANOVA	--	****	***	****	****	****

E

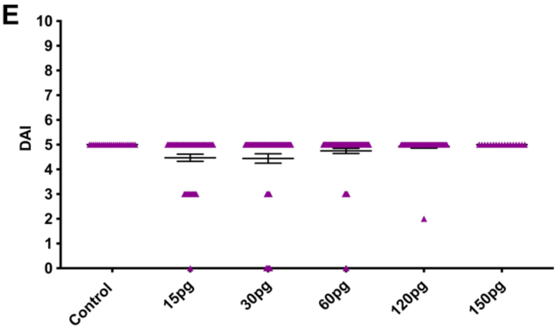

N	24	51	59	79	42	17
Mean	5	4.471	4.441	4.747	4.929	5
SD	0	1.046	1.454	0.9124	0.4629	0
SEM	0	0.1465	0.1892	0.1027	0.07143	0
ANOVA	--	ns	ns	ns	ns	ns

F

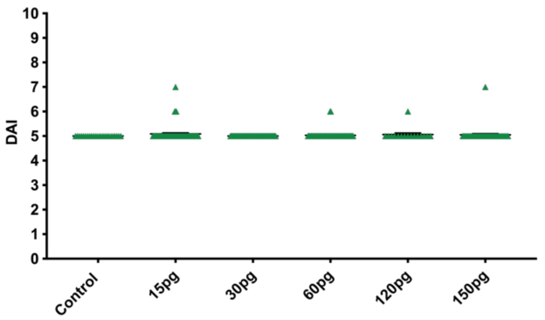

N	24	59	70	71	16	41
Mean	5	5.085	5	5.028	5.063	5.049
SD	0	0.3367	0	0.1666	0.25	0.3123
SEM	0	0.04384	0	0.01978	0.0625	0.04878
ANOVA	--	ns	ns	ns	ns	ns

G

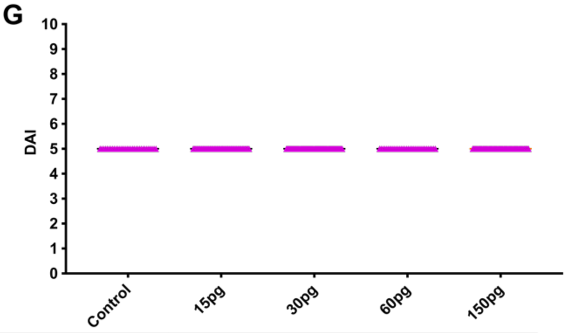

N	32	51	61	34	60
Mean	5	5	5	5	5
SD	0	0	0	0	0
SEM	0	0	0	0	0
ANOVA	--	ns	ns	ns	ns

H

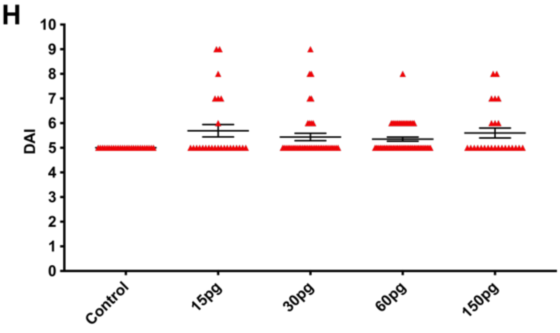

N	26	26	41	51	25
Mean	5	5.692	5.439	5.353	5.6
SD	0	1.289	0.9759	0.5941	1
SEM	0	0.2528	0.1524	0.08319	0.2
ANOVA	--	*	ns	ns	*

Figure S2: DAI scores for the microinjections done in this study. A- *Xlnog.L*, B- *Xlnog2.L*, C- *ApNL1*, D- *ApNL1ΔClip*, E- *ApNL2*, F- *ApNL2+Clip*, G- *DmTrk*, H- *DmTrk+Clip*. Controls were injected with dH_2O. Oneway ANOVA was performed comparing means with control injections. N- survived embryos with DAI phenotypes at stage 32. SD- standard deviation. SEM- standard error of mean. Error bars denote SEM. ns - >0.05, * - P ≤ 0.05, ** - P ≤ 0.01, *** - P ≤ 0.001, **** - P ≤ 0.0001.

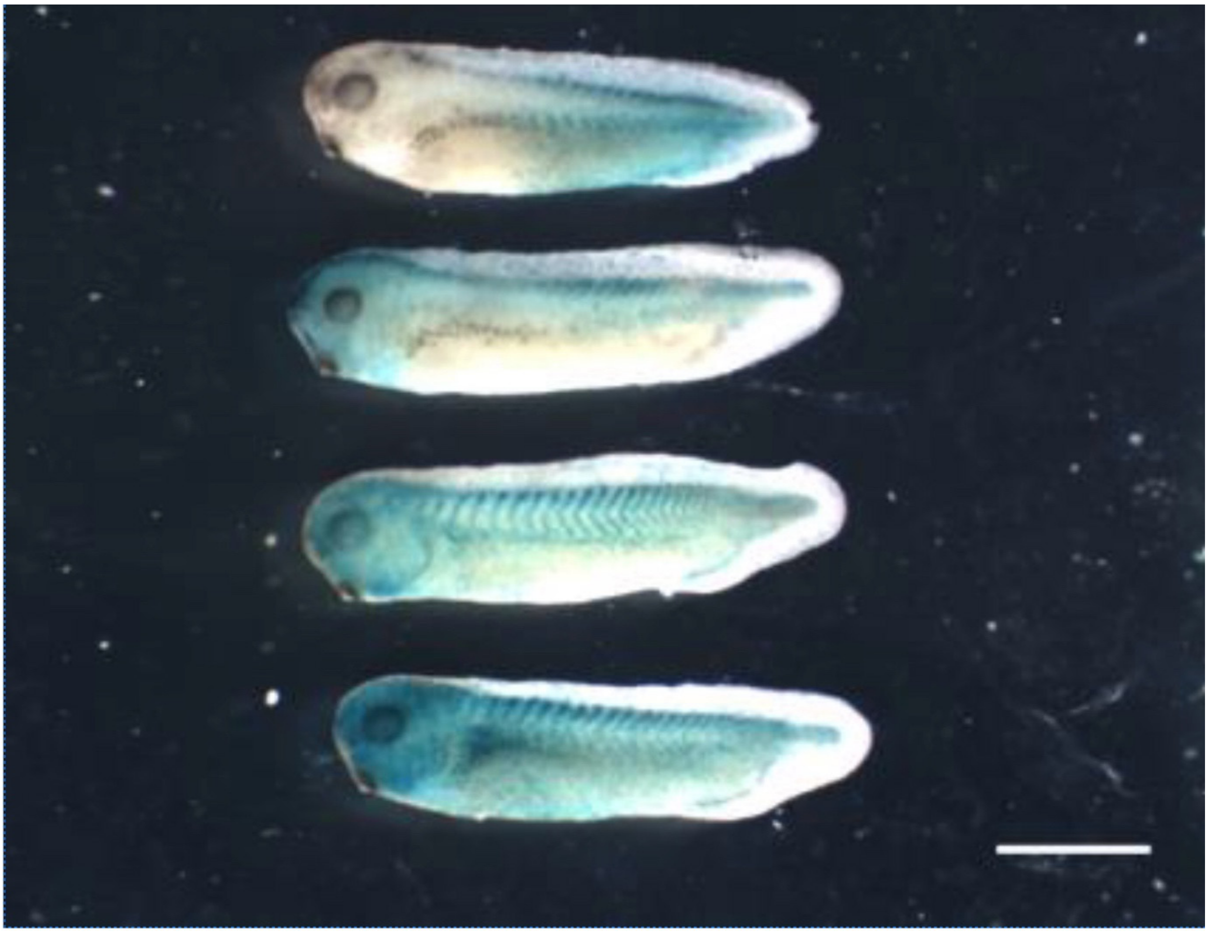

Figure S3: Stage 35 *X. laevis* embryos (DAI5) which have been injected with 230 pg of β-galactosidase mRNA, and stained for β-gal expression.

Table S1: NCBI accession numbers for the genes used in this study

**Gene**	**NCBI accession number**	
	**mRNA**	**Protein**
A. pisum NL1	XM_029486464.1	XP_029342324.1
A. pisum NL2	XM_003241931.4	XP_003241979.1
A. pisum PTTH	KY305418.1	ARM65502.1
D. melanogaster Trk	NM_057419.3	NP_476767.2
D. melanogaster PTTH	NM_001298621.1	NP_001285550.1
X.laevis Nog.L	NM_001085644.1	NP_001079113.1
X.laevis Nog2.L	NM_001095556.1	NP_001089025.1
H. sapiens Nog	NM_005450.6	NP_005441.1

Table S2: Primers used in this study

**Primer**	**Sequence**	**Source**
ApNL1-F	ACGAATGAATTCATGACTGTCAAATGG	IDT
ApNL1-R	TGCTTATCTAGATCACTCGGAGACGTC	IDT
ApNL2-F	ACGAATGAATTCATGCGGCGTTCCTTC	IDT
ApNL2-R	TGCTTATCTAGATTATTCGTCAGGCTT	IDT
ApPTTH-F	TAAGCAGAATTCATGAGACCTCAGTAC	IDT
ApPTTH-R	TGCTTATCTAGATTATTTAATAGAACA	IDT
DmTrk-F	ACGAATGAATTCATGTTTCTGCGTATA	IDT
DmTrk-R	TGCTTATCTAGACTAGTATAGCATAAC	IDT
eef1a1.L -F	CCCTGAATCACCCAGGCCAGATTGGTG	Sigma
eef1a1.L -R	GAGGTAGTCTGAGAAGCTCTCCACG	Sigma
actc1.L -F	TCCCTGTACGCTTCTGGTCGTA	Sigma
actc1.L -R	TCTCAAAGTCCAAAGCCACATA	Sigma
tbxt.L -F	AAGAATGGAAGACGAATGTTT	Sigma
tbxt.L -R	TGGGTGAGTCTGGGTGGATA	Sigma
ncam1.L -F	CACAGTTCCACCAAATGC	Sigma
ncam1.L -R	GGAATCAAGCGGTACAGA	Sigma
tubb2b.L -F	ACACGGCATTGATCCTACMG	Sigma
tubb2b.L -R	AGCTCCTTCGGTGTAATGAC	Sigma

Table S3: Fly lines used in this study

**Transgenic line**	**Genotype**	**Source**
nos-GAL4	w⁻ ; P[w+; GAL4::VP16.nos.UTR]MVB1, P{w+; UASp-GFP65C-alphatub84B}3	Bloomington Drosophila Stock Center
trkΔ9.1/CyO ; *nos*-GAL4/TM6B	trkΔ9.1/CyO;P[w+;GAL4::VP16.nos.UTR]MVB1, P{w+; UASp-GFP65C-alphatub84B}/TM6B	Johnson Lab, Monash University, Australia
UAS-trk	w⁻ ; P{w+; UAS-Trk}/TM3, Sb	Johnson Lab, Monash University, Australia
UAS-ApNL1	w⁻ ; P{w+; UAS- ApNL1}/TM3, Sb	This study
UAS-ApNL2	w⁻ ; P{w+; UAS- ApNL2}/TM3, Sb	This study
UAS-ApPTTH	w⁻ ; P{w+; UAS- ApPTTH}/TM3, Sb	This study
UAS-XlNog1	w⁻ ; P{w+; UAS- XlNog1}/TM3, Sb	This study
trkΔ9.1/CyO ; UAS-Trk/TM6B	trkΔ9.1/CyO ; P{w+; UAS- Trk}/TM6B	Johnson Lab, Monash University, Australia
trkΔ9.1/CyO ; UAS-ApNL1/TM6B	trkΔ9.1/CyO ; P{w+; UAS- ApNL1}/TM6B	This study
trkΔ9.1/CyO ; UAS-ApNL2/TM6B	trkΔ9.1/CyO ; P{w+; UAS- ApNL2}/TM6B	This study
trkΔ9.1/CyO ; UAS-ApPTTH/TM6B	trkΔ9.1/CyO ; P{w+; UAS- ApPTTH}/TM6B	This study
trkΔ9.1/CyO ; UAS-XlNog1/TM6B	trkΔ9.1/CyO ; P{w+; UAS- XlNog1}/TM6B	This study
