# Supplementary material for: Noggin proteins are multifunctional extracellular regulators of cell signaling
Source: Genetics. 2022 Mar 31;221(1):iyac049. doi: 10.1093/genetics/iyac049 (PMC9071555; doi:10.1093/genetics/iyac049)
Supplement: iyac049_Supplemental_Mateiral_Legend [file iyac049_supplemental_mateiral_legend.docx]

**Supplemental Material Legends:**

Figure S1: Multiple sequence alignment of Noggin, Noggin-like, Trunk and PTTH proteins with human Noggin.

Figure S2: DAI scores for the microinjections done in this study.

Figure S3: Stage 35 *X. laevis* embryos (DAI5) which have been injected with 230 pg of β-galactosidase mRNA, and stained for β-gal expression.

Table S1: NCBI accession numbers for the genes used in this study

Table S2: Primers used in this study

Table S3: Fly lines used in this study
